# Interventions for the Current COVID-19 Pandemic: Frontline Workers' Intention to Use Personal Protective Equipment

**DOI:** 10.3389/fpubh.2021.793642

**Published:** 2022-02-04

**Authors:** Muhammad Irfan, Sultan Salem, Munir Ahmad, Ángel Acevedo-Duque, Kashif Raza Abbasi, Fayyaz Ahmad, Asif Razzaq, Cem Işik

**Affiliations:** ^1^School of Management and Economics, Beijing Institute of Technology, Beijing, China; ^2^Center for Energy and Environmental Policy Research, Beijing Institute of Technology, Beijing, China; ^3^Department of Business Administration, Ilma University, Karachi, Pakistan; ^4^Department of Economics (DoE), Birmingham Business School (BBS), University House, Birmingham, United Kingdom; ^5^College of Social Sciences (CoSS), University of Birmingham, Birmingham, United Kingdom; ^6^School of Economics, Zhejiang University, Hangzhou, China; ^7^Public Policy Observatory Faculty of Business and Administration, Universidad Autónoma de Chile, Santiago, Chile; ^8^School of Economics, Shanghai University, Shanghai, China; ^9^School of Economics, Lanzhou University, Lanzhou, China; ^10^School of Management and Economics, Dalian University of Technology, Dalian, China; ^11^Faculty of Tourism, Anadolu University, Eskişehir, Turkey

**Keywords:** interventions–psychosocial/behavioral, infectious diseases, frontline workers, behavioral intentions, personal protective equipment (PPE), COVID-19

## Abstract

**Background:**

Frontline workers (FLWs) are at a higher risk of COVID-19 infection during care interactions than the general population. Personal protective equipment (PPE) is regarded as an effective intervention for limiting the transmission of airborne viruses. However, research examining FLWs' intention to use PPE is limited.

**Objectives:**

This study addresses this research gap and also contributes by expanding the conceptual mechanism of planned behavior theory by incorporating three novel dimensions (perceived benefits of PPE, risk perceptions of the epidemic, and unavailability of PPE) in order to gain a better understanding of the factors that influence FLWs' intentions to use PPE.

**Method:**

Analysis is based on a sample of 763 FLWs in Pakistan using a questionnaire survey, and the structural equation modeling approach is employed to evaluate the suppositions.

**Results:**

Study results indicate that attitude, perceived benefits of PPE, and risk perceptions of the epidemic have positive influence on FLWs' intention to use PPE. In comparison, the unavailability of PPE and the cost of PPE have opposite effects. Meanwhile, environmental concern has a neutral effect.

**Conclusions:**

The study results specify the importance of publicizing COVID-19's lethal impacts on the environment and society, ensuring cheap PPE, and simultaneously enhancing workplace safety standards.

## Introduction

The novel coronavirus (SARS-CoV-2) has produced devastating effects worldwide (1, 2). Nearly every country has been seriously impacted by this epidemic (3–5). On March 11, 2020, the World Health Organization (WHO) designated coronavirus 2019 (COVID-19) a global epidemic (6). According to the WHO, frontline workers (FLWs) account for 10% of all COVID-19 clinically confirmed cases worldwide (7). FLWs had higher chances of getting COVID-19 infection compared to the general population. This higher infection rate has been ascribed largely to the lack of appropriate personal protective equipment (PPE) (8). While some evidence suggests that the type of PPE may influence their level of protection against the COVID-19 infection (9, 10), there is widespread agreement on the importance of using PPE (surgical masks, gloves, eye protection, helmets, and gowns) when caring for COVID-19 patients (11). PPE is thus a crucial component of the response to the COVID-19 pandemic.

On February 26, 2020, the Pakistani Health Ministry confirmed the first COVID-19 case in the country. Within 2 weeks, COVID-19 patients increased to 20, with Sindh province dominating the other provinces (12). The COVID-19 cases are rapidly growing, and the situation is deteriorating (13). Official data exposed that the COVID-19 positive cases reached 1,289,049, with 28,830 causalities in Pakistan (14).

Former research mainly focused on scrutinizing the environmental impacts of COVID-19 (15). In this context, the first batch of studies concentrated on COVID-19 epidemiology (16, 17). The second batch of studies recognized the essential factors that impact pandemic control (18, 19). The third batch of studies evaluated the current state of disease profiles to develop precautionary measures (20, 21), whereas the fourth batch of studies examined the effect of climatic variables on pandemic transmission (22, 23). Despite former scholars' deep interest, assessing FLWs' intentions to use PPE is of prime importance. What makes FLWs different from health workers and paramedical staff in this study is the selection of respondents. Our sample of FLWs consist of respondents from Police, Rescue emergency service, non-profit organizations, and disaster management volunteer organizations. The rationale of targeting this specific segment is that there have already been many studies conducted examining the intention of health workers and paramedic staff regarding the acceptance and use of PPE (24–27). In this vein, FLWs is the only segment which has never been considered and studied in any context before. According to the authors' best knowledge, no research has been done in the perspective of Pakistan and this study is the first of its kind to examine FLWs' intention to use PPE. Thus, the findings generated based on such a sample provide a fair representation of the FLWs. The country is equipped with fewer infrastructure and healthcare resources than developed economies (28). The country is the fifth most populous in the world (29). The WHO reports that Pakistan may become the next COVID-19 hotspot unless adequate measures are taken (30). Considering this discussion, this study investigates FLWs' intentions to use PPE in relation to the following critical questions: (i) What are the potential factors that might influence FLWs from using PPE during the COVID-19 epidemic? (ii) How do these factors manipulate the intention of FLWs to use PPE? Another reason for doing this research is to advance scholarly analysis of the COVID-19 outbreak, which other researchers have not extensively examined from the Pakistani viewpoint. To do this, three more elements have been added to the conceptual framework of the theory of planned behavior (TPB).

This study makes three distinct contributions. Firstly, we were inspired by research gaps to contribute to the existing body of knowledge by identifying and analyzing the factors influencing FLWs' intention to use PPE. Secondly, the current study has added three novel aspects to the conceptual mechanism of TPB, as these aspects were never explored as potential determinants of FLWs' intention to use PPE in any context before. Finally, though the COVID-19 cases have decreased recently in Pakistan, the country is still facing several challenges to control the outbreak, including inadequate medical equipment, the high price of PPE, and the dependence on foreign countries for importing PPE. Other emerging countries are expected to face similar issues with the COVID-19. In this respect, Pakistan's situation would be seen as a representative framework for the rest of the nations to comprehend this prodigy. Additionally, the research outcomes will help other economies develop effective guidelines for the deployment of PPE in their own territories. To summarize, the current investigation preserves unique research findings in comparison to the existing pool of literature.

## Methods

### Research Framework

Public acceptance of a product is a multifaceted procedure involving a range of elements. To comprehend the dynamic character of this process, many scholars have put forward several theoretical frameworks, i.e., reasoned action theory, social cognitive theory, self-efficacy theory, and TPB (31). Among these theories, TPB has successfully scrutinized behavior, and researchers in the healthcare area have widely used it to explain and anticipate FLWs' behavior (32). TPB specifies that individuals' behavioral intentions determine behavior. Once individuals assess the implications of their actions, the behavior is carried out, resulting in the desired outcome (33).

**Figure 1:**
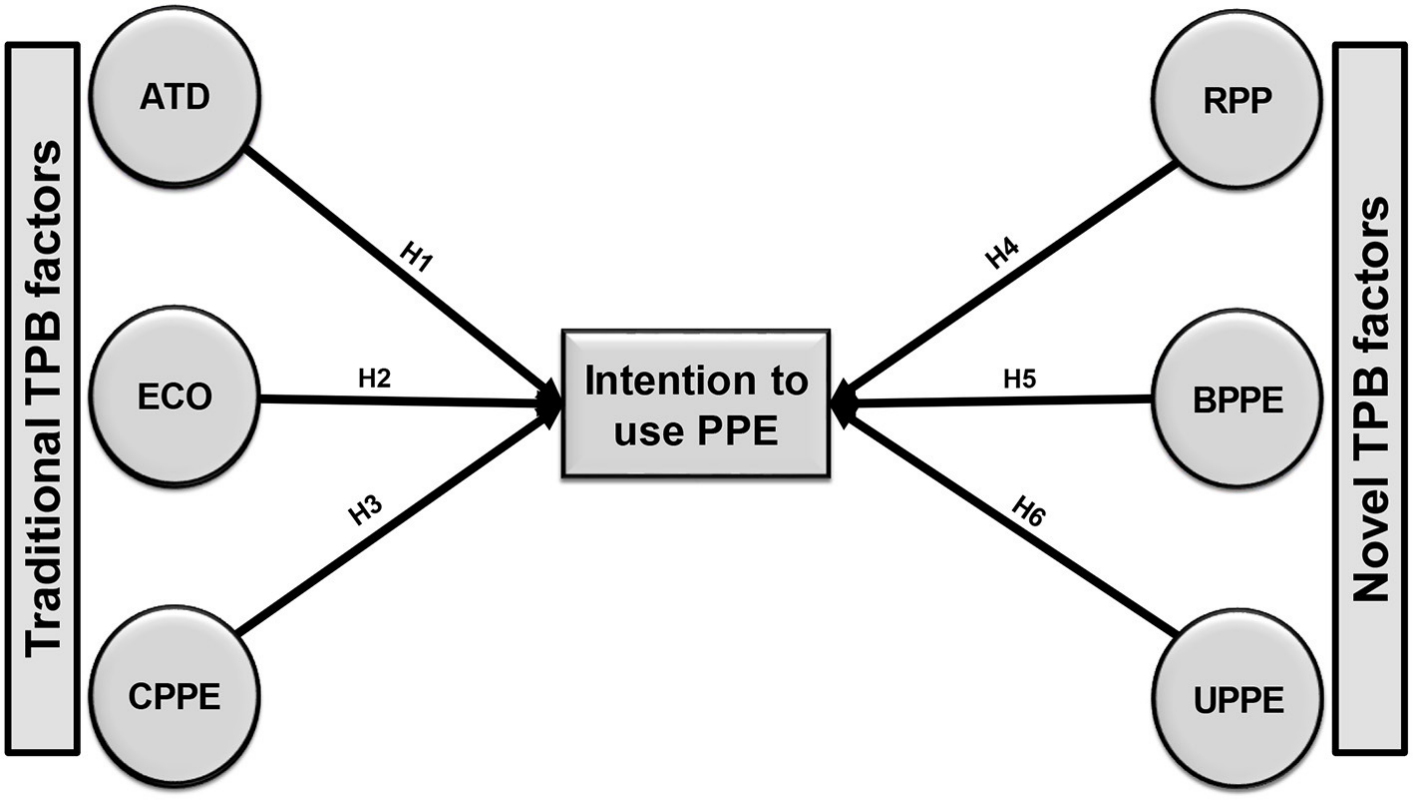
Research framework presenting the influencing factors of FLWs' intention to use PPE.

TPB has generated a considerable amount of empirical research on health behavior. Numerous researchers hypothesize that a variety of factors impact the acceptability of a specific product or service in different contexts (34). FLWs are anxious about environment, risk perceptions, safety procedures, cost, and PPE supply. Consequently, we extended the conceptual structure of TPB by introducing three new considerations. With the addition of new considerations, this research framework would help analyze FLWs' intentions to utilize PPE comprehensively. The research framework is depicted in Figure 1.

### Hypotheses Formulation

#### Attitude (ATD)

In behavioral literature, ATD is defined as FLWs' positive or negative reactions to society's health issues. Walter et al. (35) opined that public ATD during the epidemic informs mitigation methods as well as enables future epidemic preparation planning. According to previous research, there is a positive linkage between attitude and intention to use PPE. Zhang and Mu (36) concluded that people had a favorable attitude on the possibility of reducing exposure to airborne viruses with the use of PPE. Johnson and Hariharan (37) took a survey to find the attitude and behavior of people toward PPE during the Swine Flu outbreak. The results unveiled that people show high acceptance of using PPE, receiving H1NI medication, maintaining social distance, adhering to public health precautions, avoiding public transit, and keeping them away from diseased persons. These conclusions led to the formulation of 1st hypothesis as:

***H1:*** *ATD positively influences FLWs' intention to use PPE*.

#### Environmental Concern (ECO)

The level to which FLWs are aware of and committed to resolving environmental issues is termed as ECO. It is a major element influencing FLWs' decision to use PPE. Schraufnagel et al. (38) specified that FLWs with a positive ECO closely monitor the health status of others and keep a positive behavior about the use of PPE. They expect and grasp that keeping good health is a self-declared obligation. Li et al. (39) stated that current health emergencies affected the propensity to utilize PPE. Another study reported a beneficial effect of ECO on the acceptance of PPE (40). The 2nd hypothesis is devised based on these assumptions as:

***H2:*** *ECO positively influences FLWs' intention to use PPE*.

#### Cost of PPE (CPPE)

Cost is a frequently used factor to determine the financial damage associated with the purchase journey (41). Research demonstrates the negative linkage between cost and the intention to use PPE. For instance, Weiss and Palmer (42) shows that cost is the primary impediment to purchasing PPE. Kesselheim (43) examined the association between high CPPE and life-cycle management. The findings indicated that elevated costs put individuals under strain, resulting in severe health consequences. While the cost of PPE has decreased in recent years, it still remains high than the affordability of FLWs in underdeveloped countries. These assumptions enable us to formulate the 3rd hypothesis as follows:

***H3:*** *CPPE negatively influences FLWs' intention to use PPE*.

#### Risk Perceptions of the Epidemic (RPP)

RPP has a beneficial effect on public acceptance of PPE. Public acceptance increases as people become aware of their benefits to the epidemic's severity. If infection risk is high, a more rapid public response in terms of adopting preventive behaviors will occur (32). Previous research has established that risk perceptions significantly influence individuals' decisions to adopt PPE. In this respect, MacIntyre and Chughtai (44) examined the factors influencing public intention to use PPE in China and found that risk perceptions have a favorable effect on public intention. In another study, Barati et al. (45) scrutinized public behavior toward the acceptance of PPE to avoid respiratory diseases. The findings indicated that individuals are persuaded to use PPE based on the risk of contracting acute diseases. These inferences led to the formulation of the 4th hypothesis as:

***H4:*** *RPP positively influence FLWs' intention to use PPE*.

#### Perceived Benefits of PPE (BPPE)

BPPE refers to FLWs' information and knowledge of the benefits of PPE in managing and preventing the spread of infectious viral disorders (46). They believe that PPE will help prevent the virus from spreading during public gatherings and also serve as a reminder to maintain social distancing (47). Hansstein and Echegaray (48) examined the motives of using PPE among Chinese FLWs and noticed that the knowledge of climate concerns and health effects has increased in response to poor air quality in China. As a result, individuals have developed favorable beliefs toward the benefits of PPE. Based on these findings, the 5th hypothesis is stipulated as follows:

***H5:*** *BPPE positively influences FLWs' intention to use PPE*.

#### Unavailability of PPE (UPPE)

UPPE is associated with FLWs' difficulties procuring PPE (44). Previous research has established that the UPPE has a negligible effect on individuals' decisions to use PPE. Many scholars reported that UPPE negatively influences public intentions of using PPE. For instance, Tang and Wong (47) analyzed the factors affecting the intention to use PPE in the Chinese context. They opined that UPPE is a critical barrier, which negatively influences their intentions to use PPE. Similarly, MacIntyre and Chughtai (44) examined the individuals' intentions regarding PPE acceptance. Research outcomes highlighted that low acceptance is related to the UPPE, which is inefficient in preventing and treating respiratory infections. Taking these findings into account, we formulate the 6th hypothesis as follows:

***H6:*** *UPPE negatively influences FLWs' intention to use PPE*.

## Results

### Survey Region and Sample Selection

We administered an inclusive questionnaire survey in Punjab and Sindh provinces and Pakistan's federal capital territory (Islamabad) during July and August 2021. The surveyed respondents belonged to Federal Police, Rescue 1122 emergency service, Elite Police, and disaster management volunteer organizations (Aman Foundation, Green Crescent Foundation, and Edhi Foundation). Department of Federal Police is located in the Federal capital Islamabad. Rescue 1122 is the emergency service available in the Punjab province of Pakistan. Elite Force is a special branch of Punjab Police involved in risky operations. All the three stated disaster management volunteer organizations are located in Karachi city of Pakistan's Sindh province. These are non-profit organizations working across the country. Whenever there is some disaster anywhere in the country, these organizations play their volunteer role to cope with the chaotic situation. The fundamental rationale for selecting the FLWs is their frequent exposure to COVID-19 suspects and patients since FLWs have difficulties with social distancing measures. One of the reasons for the sample selection is that Islamabad is the capital city containing individuals belonging to all provinces, thus providing a heterogeneous population mix. Further, Punjab is the most populous province and involves more law enforcement agents to deal with COVID-19 containment. Besides, most welfare organizations and disaster management volunteer organizations operate from the Karachi city of Sindh province. Questionnaires were delivered to 950 FLWs, and a complete description of all questionnaire components was supplied to them (see Appendix A). A total of 763 responses were gathered, representing 80.15% of the total responses.

### Demography of the Participants

**Figure 2:**
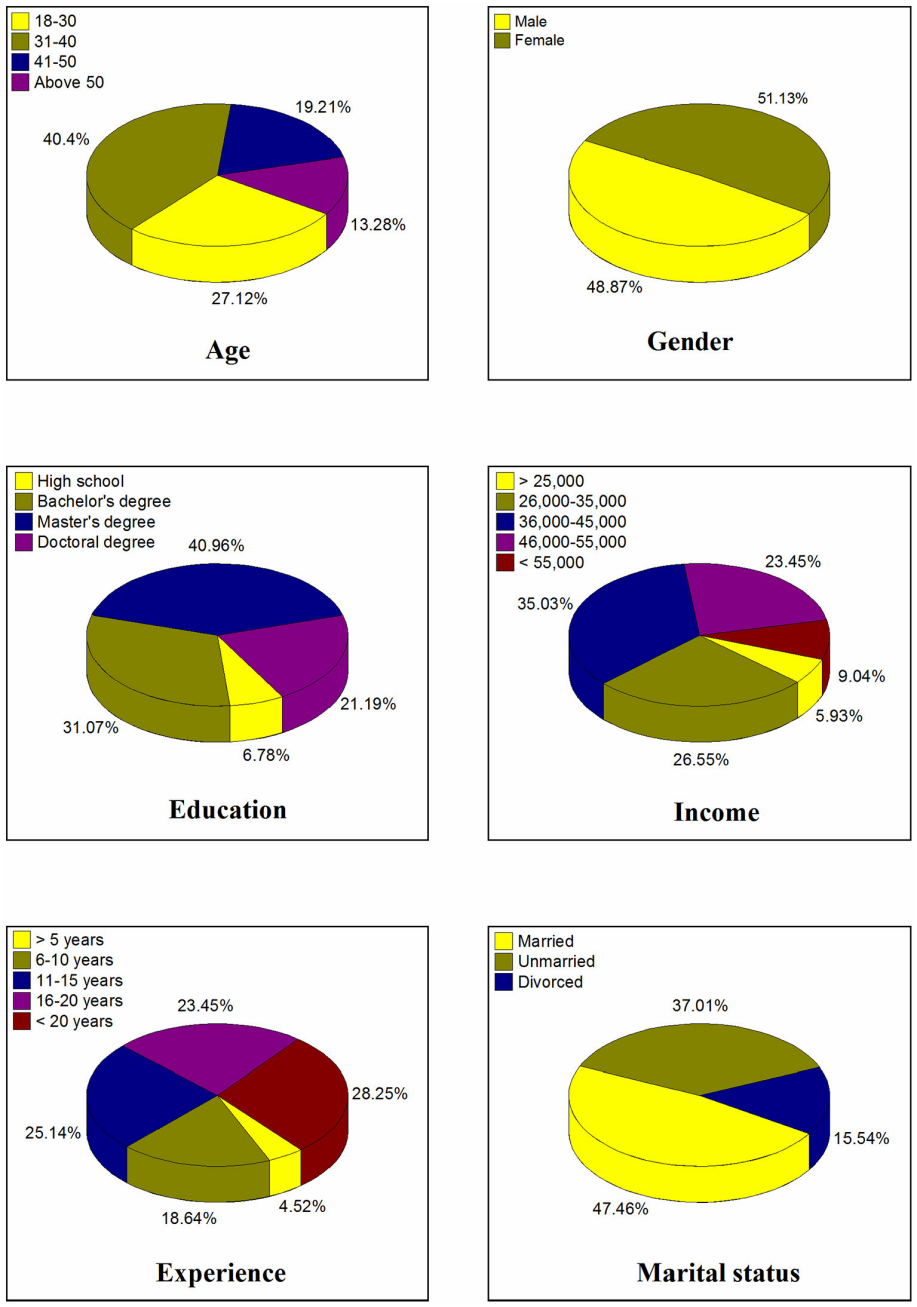
Demographic attributes of FLWs.

Figure 2 represents the demographic attributes of the participants. The lower-middle age group (40.4%) accounted for the largest proportion of participants in the survey. Females were 51.13% compared to males (48.87%) in our sample. 35.03% of the participants belonged to the middle-income class, with earning between Pakistani rupees (PKR) 35,001 and 45,000 each month. Additionally, we classified participants according to their educational degrees. 40.96% of them hold a master's degree. Most of the participants (47.46%) were married, and 28.25% had more than 20 years of professional experience.

### Statistical Summary and Discriminant Validity Analysis

The authors used SPSS and AMOS software to analyze the data and proposed hypotheses. All items were assessed on a 5-point Likert scale, as 1 indicating “strongly disagree” and 5 indicating “strongly agree.” Correlation analysis was conducted to ascertain the relationship among constructs. Discriminant validity was verified by employing the average variance extracted (AVE) square root. Discriminant validity is validated by the results, as the AVE values are greater than their association with other constructs (32). We further confirmed the discriminant validity, as all constructs' AVE values are greater than their maximum shared variance (MSV) values. Next, we looked into convergent validity by utilizing AVE values. The analysis indicated that the AVE values for all constructs were higher than 0.50, specifying that all constructs retained a minimum of 50% of the variance. The findings are reported in Table 1. Item's reliability was determined using Cronbach-α. The outcomes verified reliability as the values of Cronbach-α exceeded the minimum acceptable level of 0.70 (49). Next, we performed composite reliability (CR) test to ascertain the items' consistency across all constructs. The findings indicated that the CR values exceeded the minimum permissible value of 0.70 (50). Table 2 summarizes the results.

**Table 1:** Correlation, convergent, and discriminant validity findings.

**Variables**	**Mean**	**Std. Dev**	**ECO**	**BPPE**	**ATD**	**RPP**	**CPPE**	**UPPE**	**ITU**	**AVE**	**MSV**
ECO	3.630	0.590	**(0.712)**							0.507	0.125
BPPE	2.811	1.509	0.270	**(0.822)**						0.676	0.276
ATD	3.324	0.154	0.353	0.525	**(0.754)**					0.569	0.276
RPP	3.919	0.574	0.293	0.471	0.367	**(0.859)**				0.738	0.289
CPPE	2.603	0.661	0.170	0.416	0.305	0.538	**(0.781)**			0.609	0.524
UPPE	2.906	1.563	0.343	0.176	0.330	0.230	0.222	**(0.837)**		0.701	0.118
ITU	2.472	0.367	0.296	0.506	0.418	0.519	0.724	0.237	**(0.738)**	0.545	0.524

*The bold values represents the square root of AVEs*.

**Table 2:** Factor loadings of measurements model.

**Variables**	**Items**	**Standard**	**CR**	**Cronbach-α**
		**loadings**		
Attitude			0.902	0.914
	ATD1	0.551		
	ATD2	0.822		
	ATD3	0.715		
	ATD4	0.656		
	ATD5	0.910		
	ATD6	0.925		
	ATD7	0.622		
Environmental concern			0.804	0.815
	ECO1	0.730		
	ECO2	0.747		
	ECO3	0.680		
	ECO4	0.673		
Cost of PPE			0.886	0.880
	CPPE1	0.870		
	CPPE2	0.953		
	CPPE3	0.701		
	CPPE4	0.680		
	CPPE5	0.518		
Risk perceptions of the pandemic			0.933	0.927
	RPP1	0.743		
	RPP2	0.793		
	RPP3	0.947		
	RPP4	0.980		
	RPP5	0.796		
Perceived benefits of PPE			0.936	0.948
	BPPE1	0.644		
	BPPE2	0.828		
	BPPE3	0.804		
	BPPE4	0.856		
	BPPE5	0.853		
	BPPE6	0.824		
	BPPE7	0.908		
Unavailability of PPE			0.921	0.907
	UPPE1	0.732		
	UPPE2	0.790		
	UPPE3	0.906		
	UPPE4	0.858		
	UPPE5	0.869		
Intention to use PPE			0.827	0.834
	ITU1	0.656		
	ITU2	0.711		
	ITU3	0.635		
	ITU4	0.560		

### Hypotheses Results and Structural Model

**Figure 3:**
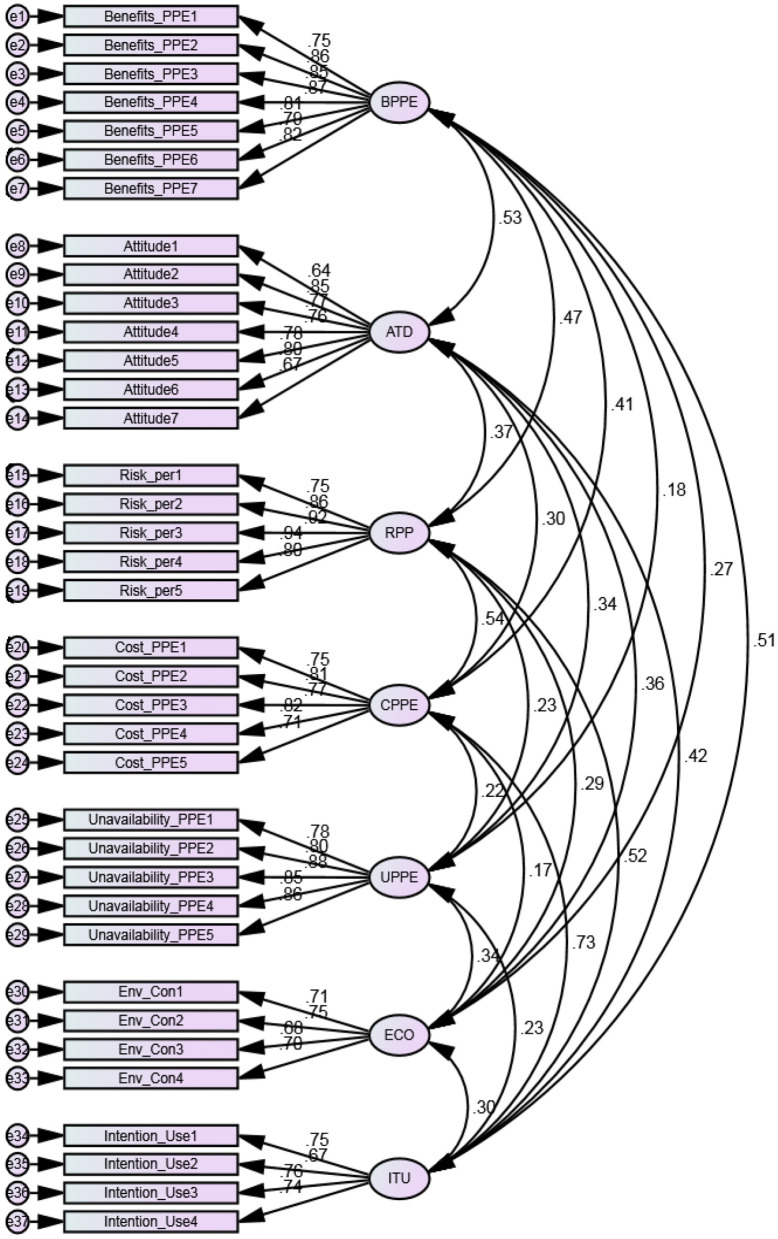
Measurement model. Authors' calculations.

Confirmatory factor analysis (CFA) was used for the purpose of model identification. The validity of the measurement model was established, as each item was loaded on its respective construct (see Figure 3). Strong *f*-values were generated for all constructs, suggesting that the relationships are linear. The *R*^2^ value of 0.68 was more than the suggested threshold of 0.35 (51), indicating a substantial interpretation. We further test the multi-collinearity in the proposed model linear regression analysis. The analysis indicates that the model is free from multicollinearity since the variance inflation factor (VIF) values are within the suggested range (52). The results are presented in Table 3.

**Table 3:** Hypotheses' findings.

**Hypotheses**	**Hypotheses paths**	***β*** **-value**	***f*** **-value**	**Result**	**VIF**	***R^2^***
*H1*	ATD → ITU	0.10^**^	216.6^***^	Accepted	1.634	0.68
*H2*	ECO → ITU	0.59	367.5^***^	Rejected	1.871	
*H3*	CPPE → ITU	−0.05^**^	120.4^***^	Accepted	1.783	
*H4*	RPP → ITU	0.09^*^	204.8^***^	Accepted	1.376	
*H5*	BPPE → ITU	0.20^**^	229.4^***^	Accepted	1.282	
*H6*	UPPE → ITU	−0.01^***^	108.6^***^	Accepted	1.809	

*** *p < 0.01*,

** *p < 0.05, and*

* *p < 0.1*.

**Figure 4:**
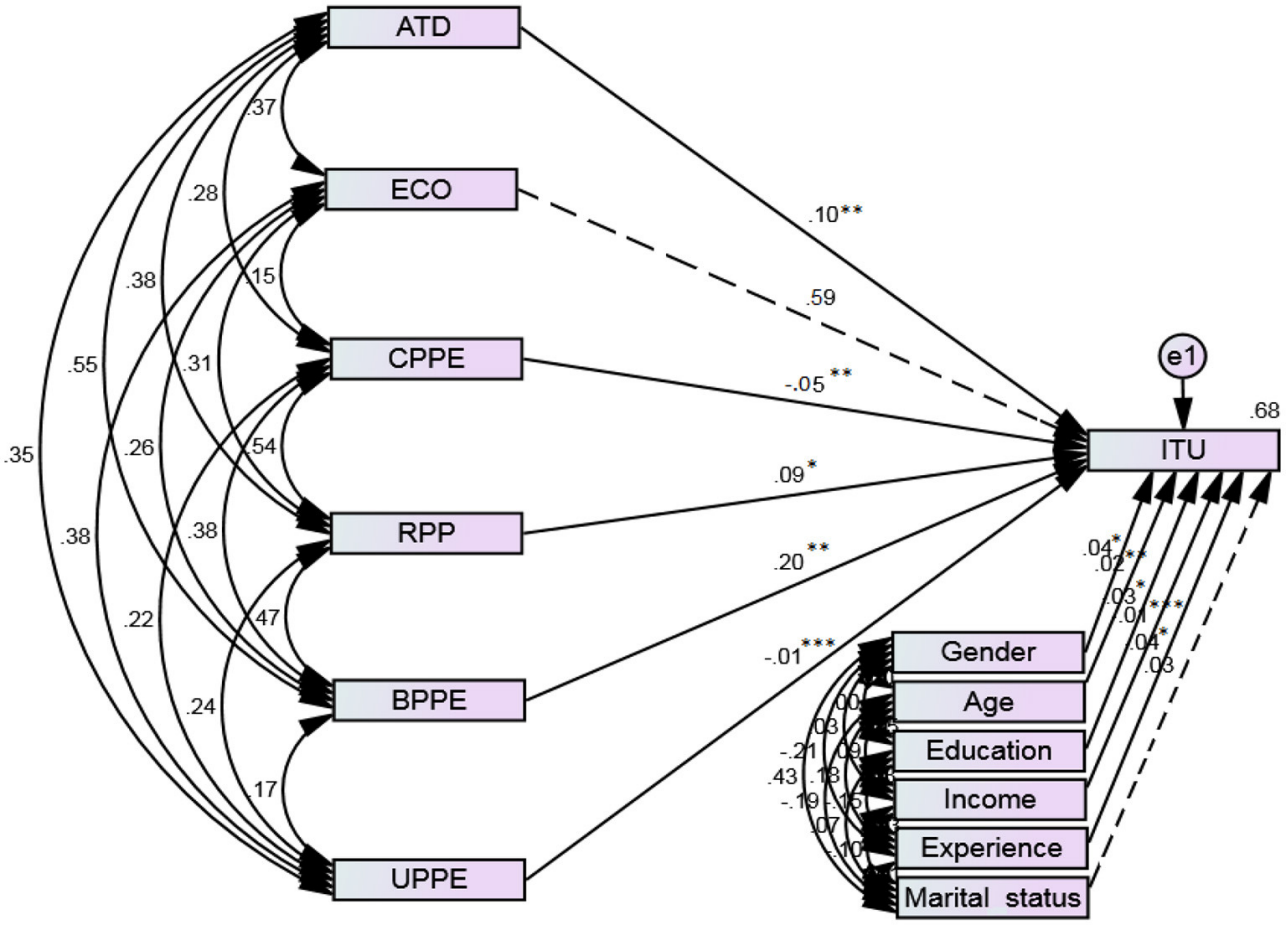
Schematic depiction of SEM. Authors' calculation. ***p < 0.01, **p < 0.05, *p < 0.1. Significant values are indicated by continuous lines, whereas insignificant values are indicated by dashed lines. CFI = 0.990, NFI = 0.959, IFI = 0.991, TLI = 0.979, GFI = 0.981, RMSEA = 0.029, *X*^2^/df = 1.290. Authors' calculations.

The schematic diagram of SEM is shown in Figure 4. We tested the influence of critical factors on FLWs' intention to use PPE using path analysis. Additionally, various fitness tests were performed to guarantee that the data was correctly fitted to the structural model. The results revealed that the values of all fit indices are according to the recommended criteria (53). The structural paths of the constructs, such as ATD (*H1*; β = 0.10, *p* < 0.01), RPP (*H4*; β = 0.09, *p* < 0.05), and BPPE (*H5*; β = 0.20, *p* < 0.01) stipulate that ATD, RPP, and BPPE significantly influence FLWs' intention to use PPE. Thus, we accepted hypotheses 1, 4, and 5. The constructs CPPE (*H3*; β = −0.05, *p* < 0.01) and UPPE (*H6*; β = −0.01, *p* < 0.001) negatively affects FLWs' intention to use PPE. Accordingly, we accepted hypotheses 3 and 6. Contrary to the formulated supposition, the structural path failed to support hypothesis 2 (*H2*; β = 0.59), because the construct “ECO” does not have a significant influence on FLWs' intention to use PPE and thus rejected (see Table 3).

## Discussion

Results supported the first hypothesis that ATD positively influences FLWs' intention to use PPE, implying that FLWs acquainted with the COVID-19 epidemic possess a greater propensity to use PPE. Zhang and Mu (36) concluded that people exhibit an optimistic attitude that PPE may help minimize the risk of viral respiratory infections. In the same vein, Johnson and Hariharan (37) uncovered a favorable impact of attitude on the intention to use PPE. The results of these studies comply with our findings. Due to the current COVID-19 outbreak, most FLWs recognize that PPE can assist reduce disease transmission and aid in managing health dilemmas. In Pakistan, knowledge of the new SARS-CoV-2 virus is increasing, which will have a greater impact on FLWs' intention to use PPE in the future. However, in some under-developed countries, people do not have adequate knowledge about the severity and the consequences of the pandemic. Consequently, they are less concerned about the viral infections and possess an unfavorable attitude toward PPE usage. This aspect is in line with the study of (54), as they found that 52% of the nurses in their sample neglected to use PPE.

Literature (40) identified that ECO positively influences intentions to use PPE. We predicted a similar pattern among Pakistani FLWs as well. However, the current study's findings have a negligible effect. A possible reason might be linked to the intentions for which FLWs use PPE. Unlike economies that focus on combating climate change and contribute actively to improving the environment (55, 56), Pakistani people attach low priority to ecological problems during purchase decisions (57–59). Another reason for this behavior is the lack of an effective policy structure. The government barely seeks to encourage residents to become conscious of environmental issues, their obligations, and active participation in environmental improvement. On the flip side, Ambigapathy et al. (60) noticed that, as the COVID-19 continues to evolve, the understanding of the pandemic is increasing among the general practitioners in Malaysia. This understanding leads to a positive environmental concern, ultimately shaping their intention to use PPE.

The likelihood of FLWs' intention to use PPE decreases with a faith about the extra cost linked with the purchase of PPE. Research findings validated the hypothesis because cost negatively influences FLWs' intention to use PPE. Previous studies have established that cost has a detrimental effect on accepting new developments in the health industry (43). In this perspective, Weiss and Palmer (42) revealed that cost influences FLWs' decision to utilize PPE. One possible explanation is that PPE is affordable in developed countries than in Pakistan. Therefore, a middle-income FLW in Pakistan is unable to pay the hefty expenses and is hesitant to buy.

Our findings reveal that RPP positively influences FLWs' intention to use PPE. Former research has established the critical impact of risk perceptions in determining public behavior during epidemics (45, 61), which is consistent with our findings. Ahmad et al. (32) examined the perception-based elements that influence individuals' intentions to endorse COVID-19 prevention measures. The findings specified that risk perceptions significantly affect individuals' behavior to undertake outbreak control measures. This shows that improving awareness of the infection's seriousness, propensity, and lethality will increase their willingness to pursue epidemic preventive solutions. Hamamura and Park (62) contrasted PPE usage by Chinese, Japanese, and American respondents. The study concluded that Chinese and Japanese individuals utilize PPE more frequently than Americans. The likely reasons that may motivate FLWs to use PPE include a perceived risk of contracting the novel epidemic and preventing viral infections. The more acute FLWs' impressions of the pandemic's fatal elements are, the easier it will be to influence their decision to use PPE. On the contrary, Izhar et al. (63) conducted a survey in Pakistan to examine the risk perceptions of COVID-19 and satisfaction with preventive measures among maternity care providers in Pakistan. The authors obtained some contrasting findings, as the risk perception of the pandemic was low among their sample. These respondents opined that COVID-19 is less contagious than tuberculosis, flu, and food poisoning. One major reason for this behavior might be that Pakistan is a developing country where tuberculosis is rampant and safe drinking water is not readily available.

Results further specify that BPPE significantly influences FLWs' intention to use PPE. These findings support earlier researches in which scholars identified that people make purchases by looking the advantages of the goods they select to buy (64, 65). FLWs agree to use PPE by recognizing the benefits associated with its use (46). One possible explanation is that, as Pakistani FLWs' understanding of environmental and health issues is growing, they are mounting positive beliefs toward PPE as a means of resolving these issues. On the other hand, certain barriers, including lack of awareness, social norms, self-effectiveness, impede PPE usage intentions. Besides, lack of advertisement form government on the comprehensive benefits of PPE usage during the pandemic also impact public BPPE.

The research findings indicate that UPPE has a detrimental effect on FLWs' intention to use PPE, which is consistent with the previous research of (44). Among the various factors discouraging FLWs from using PPE are the complexity and efforts associated with getting PPE in the specific FLWs' workplace region. In addition, because PPE acceptance and usage are still in their infancy in the country, FLWs are hesitant to adopt them. The convenience and availability of PPE would serve as important dynamics in fostering more trust in PPE usage. The previous understanding of using PPE may affect the FLWs' intentions in a manner that a pleasant experience (in the form of easy access) permits the acceptance of PPE, whereas a bad experience leads to rejection.

## Conclusions

This study assesses occupational safety behavior by assessing the factors that influence the intentions of Pakistani FLWs to use PPE. Potential motivators and deterrents of PPE usage have been recognized and evaluated. Three additional aspects have been added to the conceptual framework of TPB. The analysis is based on a sample of 763 FLWs in Pakistan using a questionnaire survey. The proposed hypotheses were analyzed using structural equation modeling. The results indicate that ATD, BPPE, and RPP have significant effects on FLWs' intention to use PPE. CPPE and UPPE have negative effects, whereas ECO shows an insignificant effect. By giving an emphasis on occupational safety behavior, this work will help as a practical guideline for governments, policymakers, and experts in the health sector by understanding the linkage among all possible factors that may influence FLWs' intentions of PPE usage.

Research results highlight the need for practitioners to be aware of the four major reasons that are making the sustainable utilization of PPE a challenging task in Pakistan. Firstly, the country has a poor economic condition. The import of expensive PPE has placed a substantial burden on the national economy. Besides, there is high uncertainty about the future supply of PPE, especially if a new outbreak happens in the country. Secondly, as evidenced by research results, FLWs in Pakistan give little priority to environmental concerns, making it very difficult to accept and utilize PPE. Thirdly, the country has an integrated social system where all ethnic groups have a shared system of meaning, language, and culture. Consequently, people mostly follow the ideas of peers, celebrities, friends, and social groups. Finally, contrary to countries where people have experienced severe outbreaks like the SARS-CoV epidemic in China, H1N1 swine flu in North America, and the Chikungunya epidemic in Italy, Pakistani people have never faced such critical circumstances before. Therefore, awareness among FLWs regarding the benefits of using PPE is low compared to these countries. The use of print and electronic media to stress COVID-19's deadly characteristics might be handy in this respect. Due to less supply, PPE is costly in the country, and pharmacies demand exorbitant rates. The government should ensure that PPE is available at a fair price, give financial assistance, and conduct frequent pricemonitoring.

Though the results of this study are generally consistent with the theoretical predictions and have important implications for practitioners, the present work is not without limitations. Firstly, the questionnaire survey has missed a principal fraction of FLWs, i.e., Health workers, Paramedic staff, etc. Potential researchers can make their survey more representative by including this fraction in subsequent studies. Secondly, the data collection is carried out only in the federal capital and two provinces of the country without considering less developed and rural regions. Socio-economic characteristics, such as education, and income, differ considerably between urban and rural regions. This limitation can be overcome by including rural FLWs as participants in future studies. Finally, the linkage between attitude and perceived benefits of PPE was not found in the current study. Scholars can tackle this issue by directing studies to see this vital linkage.

## Data Availability Statement

The raw data supporting the conclusions of this article will be made available by the authors, without undue reservation.

## Ethics Statement

This study was approved by the Ethics Committee of the Beijing Institute of Technology, China (No. 543-1). The patients/participants provided their written informed consent to participate in this study.

## Author Contributions

MI: conceptualization and writing—original draft. MI and MA: data curation. MI, SS, and ÁA-D: formal analysis. SS: funding acquisition. SS, ÁA-D, FA, AR, KA, and CI: writing—review and editing. All authors contributed to the article and approved the submitted version.

## Conflict of Interest

The authors declare that the research was conducted in the absence of any commercial or financial relationships that could be construed as a potential conflict of interest.

## Publisher's Note

All claims expressed in this article are solely those of the authors and do not necessarily represent those of their affiliated organizations, or those of the publisher, the editors and the reviewers. Any product that may be evaluated in this article, or claim that may be made by its manufacturer, is not guaranteed or endorsed by the publisher.

## Abbreviations

ATD: Attitude
AVE: Average variance extracted
BPPE: Perceived benefits of personal protective equipment
CFA: Confirmatory factor analysis
COVID-19: Novel Coronavirus disease 2019
CPPE: Cost of personal protective equipment
ECO: Environmental concern
CR: Composite reliability
FLWs: Frontline workers
ITU: Intention to use
MSV: Maximum shared variance
PKR: Pakistani rupees
PPE: Personal protective equipment
RPP: Risk perceptions of the pandemic
UPPE: Unavailability of personal protective equipment
TPB: Theory of planned behavior
VIF: Variance inflation factor
WHO: World health organization.
