# Supplementary material for: Interventions for the Current COVID-19 Pandemic: Frontline Workers' Intention to Use Personal Protective Equipment
Source: Front Public Health. 2022 Feb 4;9:793642. doi: 10.3389/fpubh.2021.793642 (PMC8855926; doi:10.3389/fpubh.2021.793642)
Supplement: Supplementary file 1 [file Table_1.pdf]

Appendix A: Survey questionnaire.

**Part 1: Demographic attributes of FLWs**						
**Gender**	Male	Female				
**Age**	18-30	31-40	41-50	Above 50		
**Income (PKR)**	> 25,00	26,000-35,000	36,000-45,000	46,000-55,000	< 50,000	
**Education**	High school	Bachelor's degree	Master's degree	Doctoral degree		
**Experience**	> 5 years	6-10 years	11-15 years	16-20 years	< 20 years	
**Marital status**	Married	Unmarried	Divorced			
**Part 2: FLWs' influencing factors affecting intention to use PPE**						
**Constructs**	**Statements**	**1**	**2**	**3**	**4**	**5**
**Attitude**						
**ATD1**	I have a favorable attitude about using PPE					
**ATD2**	I have a favorable attitude that using PPE would save me from getting an infection					
**ATD3**	When I meet with individuals, I use PPE					
**ATD4**	It is recommended to use PPE when stepping out					
**ATD5**	I believe that everyone should use PPE in public settings					
**ATD6**	I feel that using PPE during an epidemic is advantageous to society					
**ATD7**	I believe that using PPE benefits society in a positive way					
**Environmental concern**						

**ECO1**	I am concerned about the pandemic's environmental ramifications
**ECO2**	I am concerned about the spread of pandemic among my country's FLWs
**ECO3**	I am worried about the spreading of the COVID-19 outbreak in my country
**ECO4**	Climate change concerns me as a result of COVID-19's repercussions
**Cost of PPE**	
**CPPE1**	The cost of PPE is very high
**CPPE2**	Cost is a major factor when it comes to purchasing PPE
**CPPE3**	I am unable to purchase PPE because I lack sufficient money
**CPPE4**	I am unable to frequently purchase PPE
**CPPE5**	I think that buying PPE have an extra burden on my expenditures
**Risk perceptions of the pandemic**	
**RPP1**	COVID-19 is a serious outbreak
**RPP2**	FLWs who do not use PPE are prone to infection
**RPP3**	It is dangerous to venture out without using PPE
**RPP4**	I am more secure at public meetings after using PPE
**RPP5**	It is prudent to take preventative steps during the pandemic
**Perceived benefits of PPE**	
**BPPE1**	PPE usage is an excellent preventive strategy

**BPPE2**	PPE usage safeguards health
**BPPE3**	PPE usage lowers the likelihood of contracting an infection
**BPPE4**	PPE usage minimizes the likelihood of inhaling unclean air
**BPPE5**	PPE usage will help me avoid exposure to the new SARS-CoV-2 virus
**BPPE6**	I have no qualms about venturing out after using PPE
**BPPE7**	PPE usage protect people against viral infections
**Unavailability of PPE**	
**UPPE1**	There is unavailability of PPE in the country
**UPPE2**	In my opinion, the country lacks a sufficient stock of PPE
**UPPE3**	I am having problems acquiring PPE
**UPPE4**	The unavailability of PPE demotivates me to use PPE
**UPPE5**	I am unable to use PPE because of a lack of availability of PPE
**Intention to use PPE**	
**ITU1**	The outbreak encourages me to use PPE
**ITU2**	I intend to increase my expenditure on PPE
**ITU3**	I strongly recommend others to use PPE
**ITU4**	In general, I intend to use PPE
